# Human Sensorimotor Cortex Reactivates Recent Visuomotor Experience during Awake Rest

**DOI:** 10.1523/ENEURO.0134-25.2025

**Published:** 2025-04-25

**Authors:** Kenji Ogawa, Yuxiang Yang, Huixiang Yang, Fumihito Imai, Hiroshi Imamizu

**Affiliations:** ^1^Department of Psychology, Graduate School of Humanities and Human Sciences, Hokkaido University, Sapporo, Hokkaido 060-0810, Japan; ^2^Cognitive Mechanisms Laboratories, Advanced Telecommunications Research Institute International, Keihanna Science City, Kyoto 619-0288, Japan; ^3^ Institute for Advanced Co-Creation Studies, Osaka University, Suita, Osaka 565-0871, Japan; ^4^ Department of Psychology, Graduate School of Humanities and Sociology, The University of Tokyo, Bunkyo-ku, Tokyo 113-8654, Japan

**Keywords:** awake reactivation, fMRI multivariate pattern analysis, visuomotor control

## Abstract

The re-emergence of task-related activation patterns during awake rest has been reported to play a role in memory consolidation and perceptual learning. This study aimed to test whether such reactivation occurs in the primary sensorimotor cortex following a visuomotor task. During functional magnetic resonance imaging (fMRI) scanning, 42 healthy participants (13 women and 29 men) learned visuomotor tracking, while a rotational perturbation was introduced between the cursor position and joystick angle. This visuomotor task block was interleaved with a control block, during which participants passively viewed a replay of their previously performed cursor movements. Half of the participants used their right hand, whereas the other half used their left hand to control the joystick. Resting-state scans were acquired before and after the visuomotor task sessions. A multivariate pattern classifier was trained to classify task and control blocks and was then tested on resting-state scans collected before and after the task session. Results revealed a significant increase in the number of volumes classified as “task” during post-task rest compared with pre-task rest, indicating re-emergence of task-related activity. Representational similarity analysis also showed a greater similarity to task-related patterns during the post-task rest period. Furthermore, this effect was specific to the left primary sensorimotor cortex contralateral to the hand used and significantly correlated with motor improvement following rest. Our findings reveal the reactivation of recent task-related experience in the primary sensorimotor cortex.

## Significance Statement

Previous studies have reported the re-emergence of task-specific activity patterns during awake rest. In this study, we aimed to determine whether such reactivation occurs in the primary sensorimotor cortex following a visuomotor task, potentially supporting offline memory consolidation. Our results showed a significant increase in task-classified brain volumes during the post-task rest period compared with the pre-task period, indicating a re-emergence of task-related activity. Notably, this effect was specific to the left primary sensorimotor cortex contralateral to the hand used for the task and was significantly correlated with motor performance following the rest period. These findings provide evidence for the reactivation of recent task-related patterns after a visuomotor task, which may support memory consolidation processes.

## Introduction

The brain generates spontaneous activity even in the absence of specific tasks or external inputs (Raichle et al., 2001; Fox and Raichle, 2007). Research has shown that this spontaneous activation during rest is modulated by recent experiences, contributing to the reorganization and consolidation of memories (Tambini and Davachi, 2019). Animal studies suggest that resting-state (RS) brain activity reflects a prior distribution in visual perception, helping to construct an internal model of the environment (Berkes et al., 2011). In humans, functional magnetic resonance imaging (fMRI) studies have demonstrated changes in RS activity within the early visual cortex following visual perceptual learning (Lewis et al., 2009; Guidotti et al., 2015), as well as enhanced RS functional connectivity between the hippocampus and a region of the lateral occipital complex associated with memory consolidation (Tambini et al., 2010).

Neural replay during rest after training is thought to be a mechanism for memory consolidation, reflecting cortical plasticity (Kurth-Nelson et al., 2023). Replay or reactivation in the hippocampus is considered a core component of active systems consolidation (Klinzing et al., 2019). The first empirical evidence for this phenomenon came from recordings of neuronal assemblies in rodents during sleep, showing that the hippocampus autonomously reactivates the same neuronal patterns engaged during prior experiences (Pavlides and Winson, 1989). Subsequent studies have demonstrated that a similar “replay” or reactivation also occurs in the visual cortex of rodents, not only during sleep (Ji and Wilson, 2007) but also during awake rest. This spontaneous reactivation, which can last for several minutes following repeated visual stimulation, is believed to facilitate short-term memory and contribute to long-term perceptual learning (Han et al., 2008).

In the motor domain, a recent invasive study recorded the brain activity of a participant with tetraplegia who completed a novel motor task and found that neural signals recorded overnight replayed the learned target sequences in the motor cortex (Rubin et al., 2022). Another invasive study involving two participants with tetraplegia and intracranial recordings revealed replay of activity for a learned motor sequence in the motor cortex during awake rest (Eichenlaub et al., 2020). These invasive brain recording studies suggest that offline replay of neural firing during sleep or wakefulness plays a role in memory consolidation. Additionally, noninvasive human studies using fMRI have reported widespread enhanced activation of sensorimotor networks during awake rest following training (Albert et al., 2009; Vahdat et al., 2011; Sami et al., 2014; Lin et al., 2018). Similar findings have been observed using electroencephalography (Wu et al., 2014; Gentili et al., 2015), with these reactivations being linked to performance improvement through offline learning (Gregory et al., 2014; Manuel et al., 2018). A recent study using both structural MRI and univariate fMRI analysis demonstrated that increased activity in the hippocampus and precuneus during quiet rest periods interleaved with practice predicted performance improvements after the rest periods (micro-offline gains) while participants learned a novel motor sequence (Jacobacci et al., 2020). Another recent study in humans, using multivariate pattern analysis of fMRI data, reported reactivations in the hippocampus and striatum during awake rest following motor sequence learning (King et al., 2022). However, it remains unclear whether neural populations related to prior sensorimotor experience are reactivated in the primary sensorimotor cortex and whether such reactivation is associated with offline improvements in behavioral performance.

This study aimed to test whether such re-emergence of activation occurs in the human sensorimotor cortex following a visuomotor task. In our experiments, participants performed continuous manual visuomotor tracking inside an MRI scanner, and we measured brain activity using fMRI during both the task period and the resting periods before and after the task. Using multivoxel pattern analysis (MVPA), we first examined whether brain activity patterns during rest resembled those observed during the preceding motor task (i.e., reactivation). Additionally, we investigated whether such reactivation facilitated behavioral performance after the task.

## Materials and Methods

### Participants

Participants included 42 volunteers (13 women and 29 men) with a mean age of 22.7 years (range, 20–34 years). The sample size was determined prior to data collection based on our previous study, which used MVPA with two groups of participants to compare neural representations in the primary sensorimotor cortex (Ogawa et al., 2019). Half of the participants (*n* = 21, 6 women) used their right hand (right-hand group), whereas the other half (*n* = 21, 7 women) used their left hand (left-hand group) to control a joystick in the MRI scanner. One participant (male) from the left-hand group was excluded from the analysis because he did not move the cursor frequently during the task period. All participants were right-handed, as assessed using a modified version of the Edinburgh Handedness Inventory (Oldfield, 1971) adapted for Japanese participants (Hatta and Nakatsuka, 1975). Written informed consent was obtained from all participants in accordance with the Declaration of Helsinki. The experimental protocol was approved by the local ethics committee.

### Task procedures

**Figure 1.**
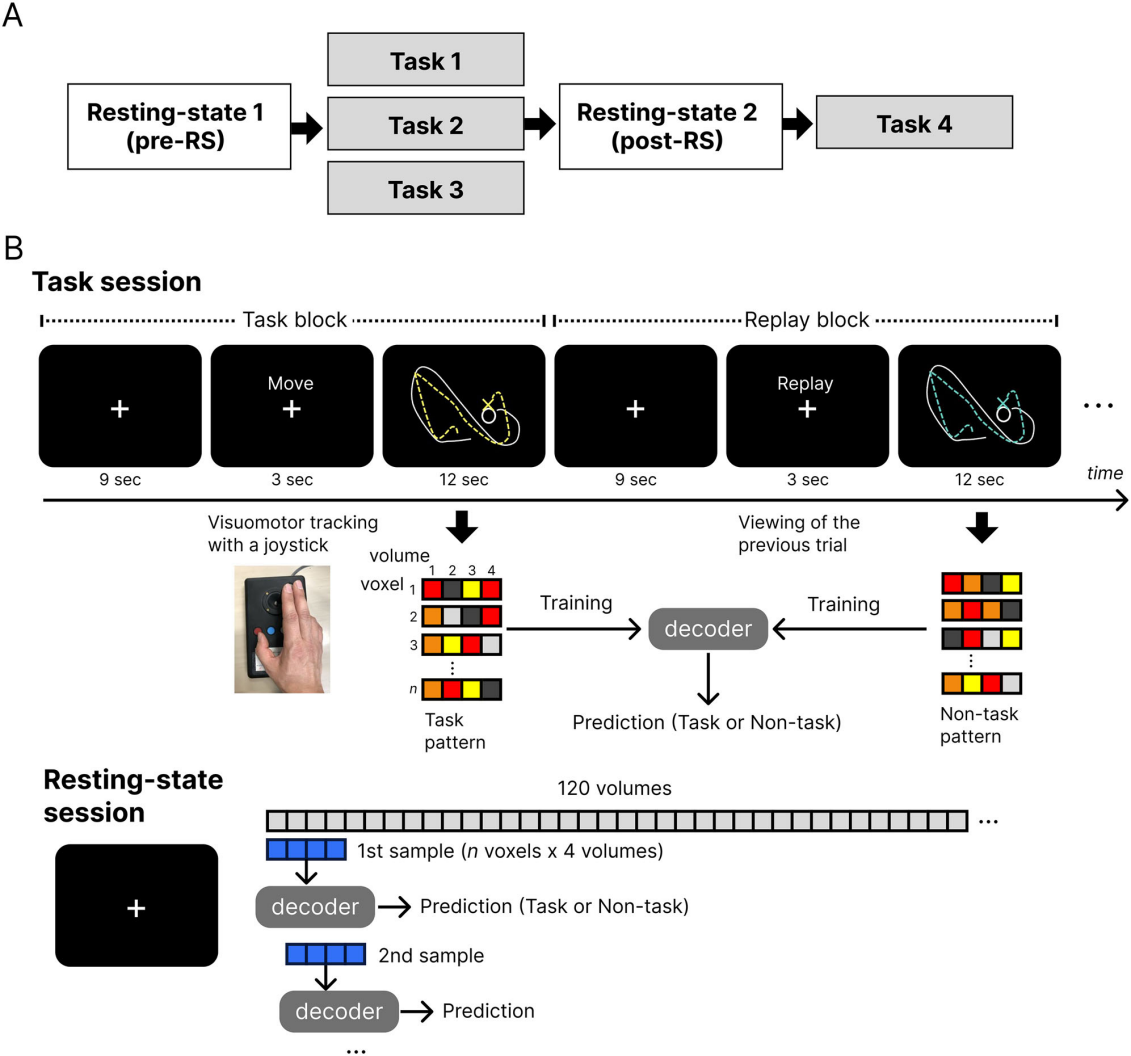
Schematic description of the experimental time course and multivoxel pattern analysis (MVPA). ***A***, Experimental schedule showing the sequence of task and resting-state (RS) scanning sessions. ***B***, Top, Example time course of a single trial within a task session, consisting of one task block followed by one replay block. Bottom, Illustration of multivoxel activity patterns during task and replay blocks within the task sessions. These were used as task and non-task templates to train the decoder. Activity patterns from three task sessions were used for training, and the decoder was then applied to pre-RS and post-RS data to test for the presence of task-related patterns during rest.

Participants underwent fMRI scanning, which consisted of four task sessions and two RS sessions—one before (pre-RS) and one after (post-RS) the first three task sessions (Fig. 1*A*). During the task sessions, participants performed a continuous visuomotor tracking task (Ogawa and Imamizu, 2013), in which a 30° rotational perturbation was applied between the joystick angle and the cursor position. In each visuomotor task block (12 s), participants were instructed to track a randomly moving target on the screen by controlling a cursor with the joystick. These task blocks were interleaved with replay blocks (12 s), during which participants passively viewed a replay of their own previously recorded cursor movements. The replay condition served as a control to isolate movement execution-related brain activity by subtracting visual processing effects. Each task session consisted of 10 task blocks and 10 replay blocks. In the RS sessions, participants were instructed to fixate on a central cross, avoid movement, stay awake, and remain relaxed. Each RS session lasted 6 min (Fig. 1*B*). Finally, the participants underwent T1-weighted anatomical scanning.

### MRI acquisition

All scans were performed using a 3 T Siemens Prisma scanner equipped with a 64-channel head coil at Hokkaido University. T2*-weighted echoplanar imaging (EPI) was used to acquire a total of 174 scans per task session and 122 scans per RS session, using a gradient EPI sequence. The first three scans within each session were discarded to allow for T1 equilibration. The scanning parameters were as follows: repetition time (TR), 3,000 ms; echo time (TE), 30 ms; flip angle (FA), 90°; field of view (FOV), 192 × 192 mm; matrix size, 94 × 94; 36 axial slices; and slice thickness, 3.0 mm with a 0.75 mm gap. T1-weighted anatomical images were acquired using an MP-RAGE sequence with the following parameters: TR, 2,300 ms; TE, 2.41 ms; FA, 8°; FOV, 256 × 256 mm; matrix size, 256 × 256; 224 axial slices; and slice thickness, 0.8 mm with no gap.

### fMRI mass-univariate analysis

Image preprocessing was performed using SPM12 software (Wellcome Department of Cognitive Neurology, http://www.fil.ion.ucl.ac.uk/spm). All functional images were initially realigned to correct for motion-related artifacts. Volume-based realignment was conducted using rigid-body transformation to minimize the squared differences between volumes. The realigned images were then spatially normalized to the Montreal Neurological Institute template using affine and nonlinear registration based on the coregistered T1-weighted anatomical images (SPM normalization procedure). Images were resampled into 3 mm isotropic voxels using sinc interpolation and subsequently smoothed with a Gaussian kernel of 6 × 6 × 6 mm full-width at half-maximum. However, images used for MVPA were not smoothed to preserve fine-grained multivoxel information (Mur et al., 2009; Kamitani and Sawahata, 2010). We performed mass-univariate analysis to identify regions significantly activated during the task blocks compared with the replay (observation-only) blocks. Both conditions were modeled as separate boxcar functions (12 s duration) convolved with the canonical hemodynamic response function in SPM. Six motion parameters from the realignment step were included as confounding covariates. We then analyzed significantly activated voxels associated with the task block compared with the replay block. Contrast images for each subject, generated using a fixed-effects model, were entered into a group-level analysis using a random-effects model conducted separately for each group. Activation was reported with a threshold of *p* < 0.05, corrected for multiple comparisons using familywise error correction, with an extent threshold of 15 voxels. A conjunction analysis was also conducted to identify commonly activated regions across both groups, using the same threshold and a logical AND operation.

### MVPA

We used MVPA to classify the task and replay activities using a spatiotemporal decoder (Guidotti et al., 2015). The classifier was based on a linear support vector machine run by LIBSVM (http://www.csie.ntu.edu.tw/∼cjlin/libsvm), with a fixed regularization parameter, *C* = 1. The region of interest (ROI) was defined anatomically as the primary sensorimotor cortex, comprising the precentral and postcentral cortices, using the automated anatomical labeling (AAL) toolbox (Tzourio-Mazoyer et al., 2002). We applied a high-pass filter with a cutoff period of 128 s to remove low-frequency drift in the fMRI signals and then *z*-normalized the signal time series within each voxel. First, we attempted to classify brain activity during the task and replay blocks using leave-one-session-out cross-validation across the first three task sessions. The activation patterns during the task and replay blocks, each consisting of four consecutive volumes in the three task sessions, were used as the spatiotemporal patterns to train the decoder (Fig. 1*B*, middle). We labeled the scanned volumes during the task and replay blocks with a temporal shift of 6 s (2 TRs), accounting for the hemodynamic delay in the fMRI signal. This analysis produced the mean classification accuracy across the three task sessions using leave-one-session-out cross-validation. Next, we investigated whether the reappearance of task-related activation patterns occurred more frequently in the primary sensorimotor cortex after the task sessions (post-RS) than before (pre-RS). As in the above analysis of the task sessions, the decoder was first trained to classify brain activity between the task and replay blocks using data from the three task sessions. This decoder was then tested on activity from the pre-RS and post-RS sessions to determine whether task-like brain activity patterns occurred during the rest session (Fig. 1*B*, bottom). This analysis used a sliding time window, in which a window equal in length to the task or replay block (four volumes) was slid across the resting scan volumes, advancing one volume per analysis.

Pattern similarity-based classification was also conducted using representational similarity analysis (RSA; Kriegeskorte et al., 2008). First, we computed the average spatiotemporal activity patterns for the task and non-task (replay observation) blocks, which served as templates for each sample. As in the previous classification analysis, spatiotemporal patterns were constructed from multivoxel activity across four consecutive volumes. We then calculated the Euclidean distance in the spatiotemporal multidimensional feature space between each activation pattern during the pre- or post-RS sessions and the task/replay templates. This analysis also employed a sliding time window: a four-volume window (corresponding to the task/replay block duration) was moved through the RS scan volumes, advancing by one volume at a time. If the RS activation pattern was closer (i.e., had a shorter Euclidean distance) to the task template than to the non-task template, it was classified as a task volume; otherwise, it was classified as a non-task volume.

## Results

### Behavioral results

**Figure 2.**
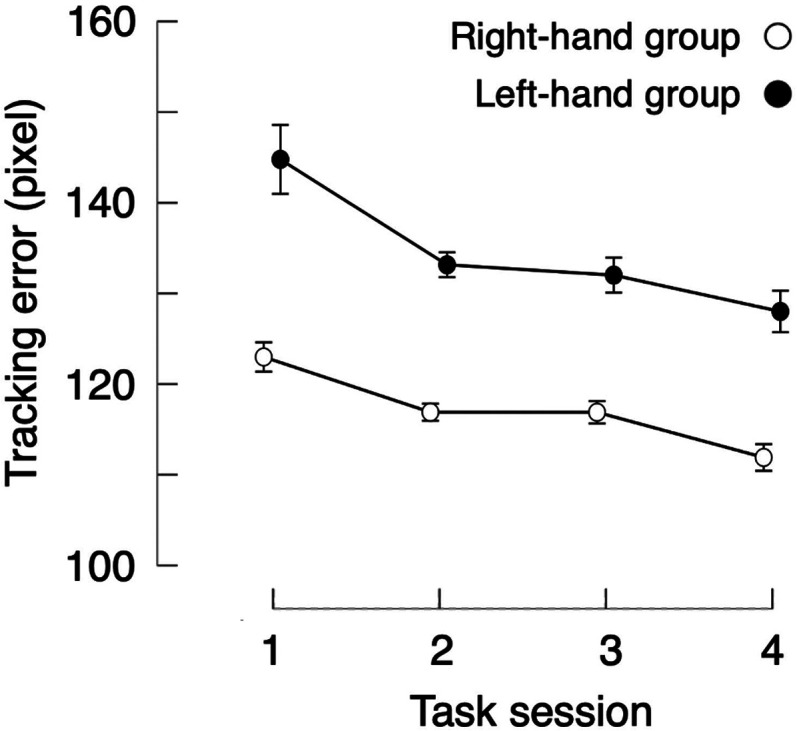
Behavioral results of tracking error. Average tracking error (in pixels) between the target and the cursor during the task sessions for the right-hand group (white dots) and the left-hand group (black dots). Error bars denote SEMs.

We conducted a mixed-effects analysis of variance (ANOVA) on the average tracking error between the right-hand and left-hand groups across the four task sessions. The results showed a significant main effect of group (*F*_(1,39)_ = 7.65, *p* = 0.01, *η_p_^2^* = 0.16) and of session (*F*_(3,117)_ = 17.19, *p* < 0.001, *η_p_^2^* = 0.31), with no significant interaction (*F*_(3,117)_ = 1.16, *p* = 0.33, *η_p_^2^* = 0.03). Post hoc comparisons revealed a significantly larger tracking error in task Session 1 compared with Session 2 (*t*_(40)_ = 4.46, *p_Bonf_* < 0.001), Session 3 (*t*_(40)_ = 4.75, *p_Bonf_*  < 0.001), and Session 4 (*t*_(40)_ = 7.01, *p_Bonf_* < 0.001), with a marginally significant difference between Sessions 2 and 4 (*t*_(40)_ = 2.55, *p*_Bonf_ < 0.1; Fig. 2).

### fMRI mass-univariate analysis

**Figure 3.**
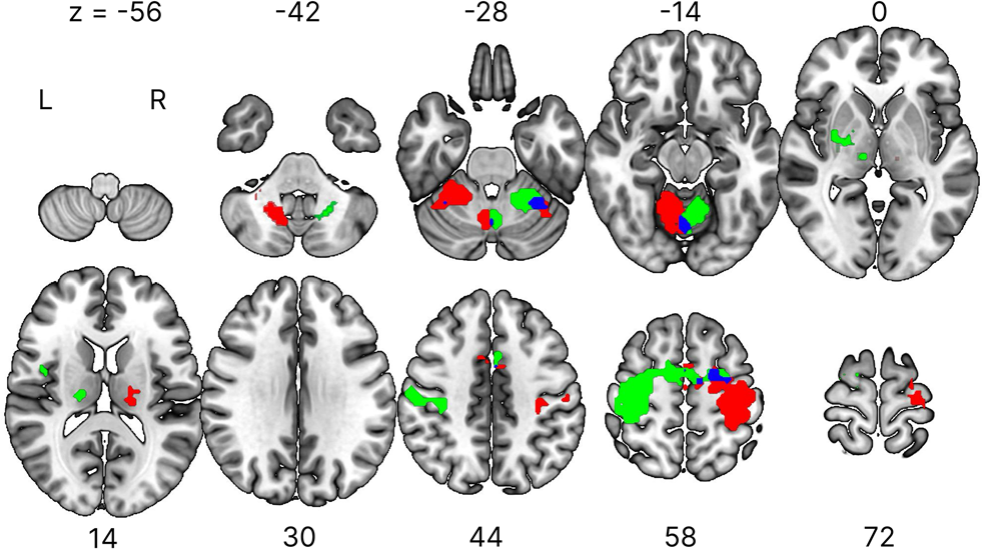
Activated areas during the task block identified via mass-univariate analysis. Brain regions showing significant activation during the task block compared with the replay block for the right-hand group (green) and the left-hand group (red). Overlapping activation between groups is shown in blue, based on a conjunction analysis (logical AND operation). Activation was reported with a threshold of *p* < 0.05, corrected for multiple comparisons using familywise error correction, with an extent threshold of 15 voxels. MNI coordinates of the activated regions are listed in Table 1. Results are displayed on horizontal brain slices, with *Z* denoting the slice location in MNI space. L, left hemisphere; R, right hemisphere.

We analyzed the activated regions of the brain using conventional mass-univariate analysis of single voxels for each group. We compared the activity between the task and replay blocks across four task sessions to identify the brain regions significantly activated during the task block. We found that the activations were mainly in the primary sensorimotor cortex and the cerebellum, either contralateral or ipsilateral to the hand used, along with small clusters in the thalamus, basal ganglia, and central operculum for both groups (Fig. 3, Table 1). Additionally, conjunction analysis revealed common activation between the groups in the medial and right parts of the cerebellum as well as the right precentral cortex.

**Table 1.** Anatomical regions, peak voxel coordinates, and *t* values of observed activation during task blocks compared with replay blocks for each group

Anatomical region	Voxels	MNI coordinates			*t*-value
		*x*	*y*	*z*	
Right-hand group					
R cerebellum	673	21	−49	−22	20.45
L precentral/postcentral gyrus	713	−30	−25	56	16.09
L thalamus	55	−12	−19	5	11.47
L cerebellum	63	−30	−58	−25	5.62
Left hand group					
L cerebellum	400	−18	−52	−22	16.35
R precentral/postcentral gyrus	845	36	−13	59	15.39
R thalamus	55	12	−19	5	15.08
L cerebellum	62	−21	−58	−46	12.43
R basal ganglia	38	21	−7	−1	5.94
R central operculum	22	42	−1	14	9.66
R cerebellum	15	30	−55	−22	5.30

Activation was reported at a threshold of *p* < 0.05, corrected for familywise error, with an extent threshold of 15 voxels. MNI, Montreal Neurological Institute; L, left hemisphere; R, right hemisphere.

### MVPA

**Figure 4.**
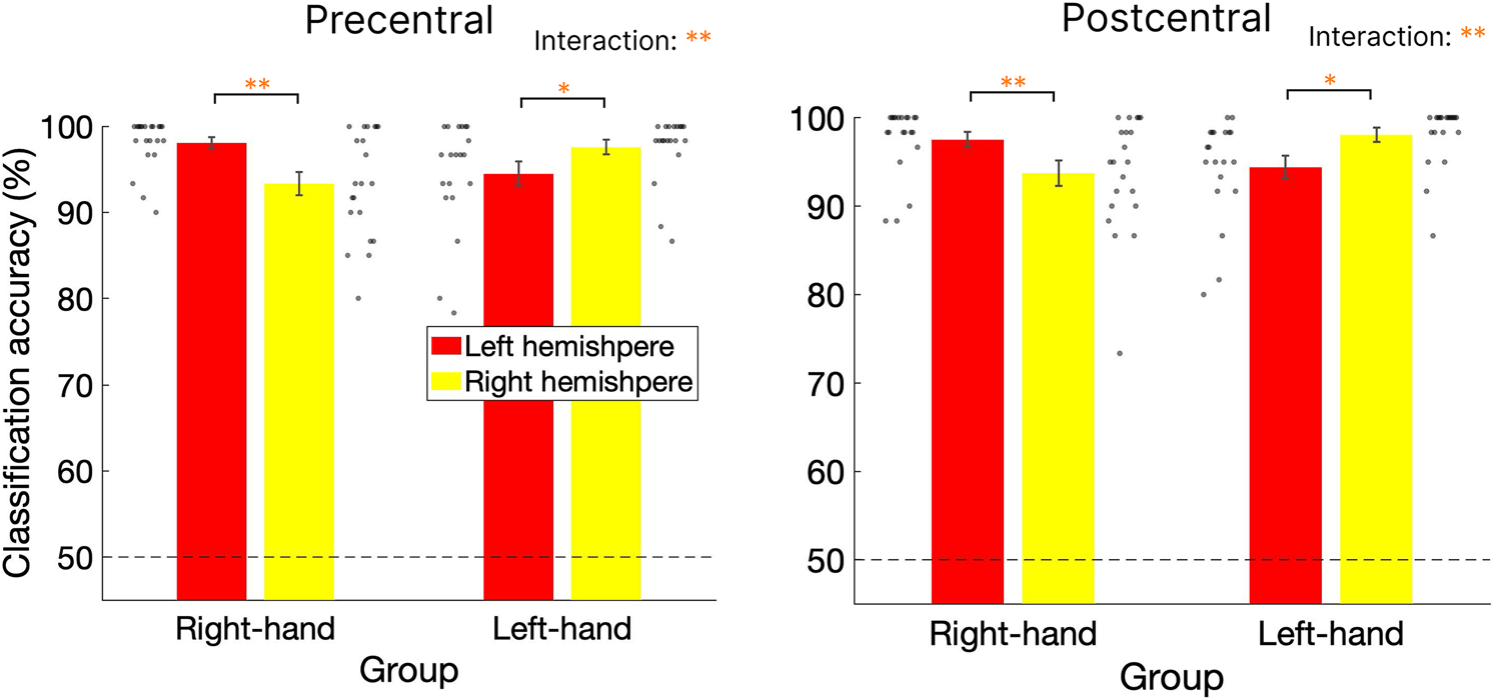
Classification results of task and replay block activity across task sessions. Classification accuracy for distinguishing task and replay (observation-only) block activity across the three task sessions in the precentral cortex (left) and postcentral cortex (right) for each group. Red and yellow bars indicate results from the left and right hemispheres, respectively. Gray dots represent individual participant data. Black dotted lines denote the chance level of classification. Error bars denote SEMs. *p < 0.05; **p < 0.005.

We first classified brain activity during the task blocks versus the replay (observation-only) blocks using leave-one-session-out cross-validation across the first three task sessions. All mean classification accuracies exceeded 90% (ranging from 93.3 to 98.1%) for both ROIs and groups and were significantly above chance (*p*s < 0.001, Cohen's *d* > 7.20). A mixed-effects ANOVA was then conducted on classification accuracy in the precentral and postcentral cortices, with hemisphere (left/right) as a within-subject factor and group (left-hand/right-hand) as a between-subject factor. In the precentral cortex, a significant interaction was found (*F*_(1,39)_ = 22.07, *p* < 0.001, *η_p_^2^* = 0.36), with no significant main effects of group (*F*_(1,39)_ = 0.06, *p* = 0.81, *η_p_^2^* = 0.002) or hemisphere (*F*_(1,39)_ = 1.01, *p* = 0.32, *η_p_^2^* = 0.025). Post hoc analysis revealed significantly higher classification accuracy in the contralateral hemisphere for both the right-hand (*t*_(20)_ = 4.46, *p* < 0.001, Cohen's *d* = 1.00) and left-hand (*t*_(19)_ = −2.36, *p* = 0.03, Cohen's *d* = 0.55) groups. In the postcentral cortex, a similar pattern was observed with a significant interaction (*F*_(1,39)_ = 19.07, *p* < 0.001, *η_p_^2^* = 0.328), with no significant main effects of group (*F*_(1,39)_ = 0.21, *p* = 0.65, *η_p_^2^* = 0.005) or hemisphere (*F*_(1,39)_ = 0.007, *p* = 0.93, *η_p_^2^* = 0.0002). Post hoc analysis again showed significantly higher classification accuracy in the contralateral hemisphere for both the right-hand (*t*_(20)_ = 3.41, *p* = 0.003, Cohen's *d* = 0.73) and left-hand (*t*_(19)_ = −2.81, *p* = 0.01, Cohen's *d* = 0.64) groups. These results demonstrate successful classification of task versus non-task patterns, with significantly higher accuracy in the contralateral sensorimotor cortex than in the ipsilateral side of the hand used (Fig. 4).

**Figure 5.**
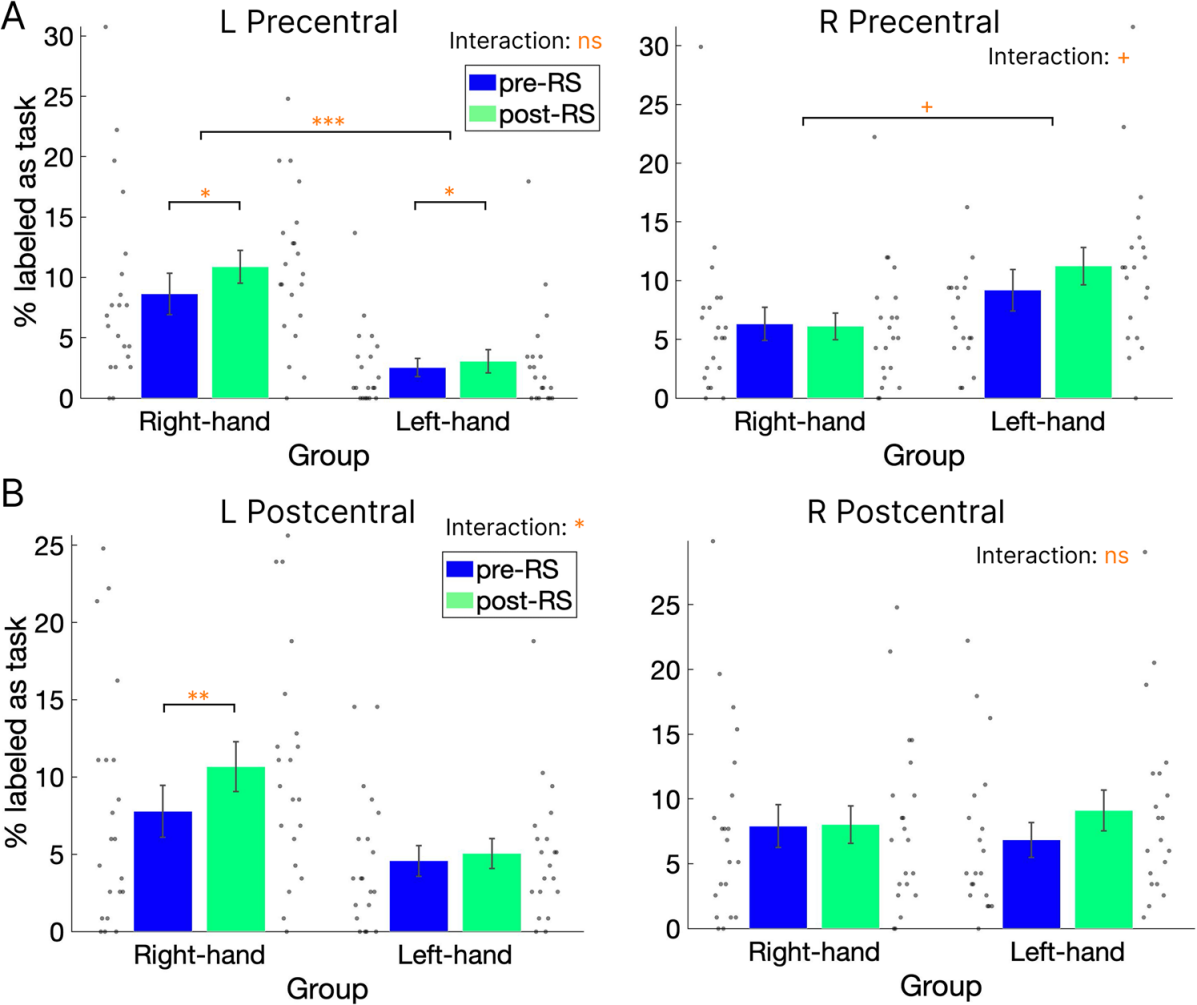
Percentage of volumes labeled as task patterns during resting-state (RS) sessions. Percentage of volumes classified as task-related patterns in the precentral (***A***) and postcentral (***B***) cortices during the pre-task RS (pre-RS, blue) and post-task RS (post-RS, green) sessions for each group. Gray dots represent individual participant data. L, left hemisphere; R, right hemisphere. Error bars denote SEMs. ns, non-significant; ^+^*p* < 0.10; *p < 0.05; **p < 0.01; ***p < 0.005.

Next, we investigated whether the re-emergence of task-related activation patterns occurred more frequently after the task sessions (post-RS) than before the task sessions (pre-RS). As in the previous analysis, the decoder was first trained to classify brain activity patterns as task or non-task using all three task sessions. This trained decoder was then applied to the pre-RS and post-RS data to determine whether task-like brain activity patterns emerged during the RS sessions. A mixed-effects ANOVA was conducted on the percentage of volumes classified as task patterns, with session (pre-RS/post-RS) as a within-subject factor and group (right-hand/left-hand) as a between-subject factor. In the left precentral cortex (Fig. 5*A*, left), a higher percentage of task-classified volumes was observed in the post-RS session than in the pre-RS session, as indicated by a significant main effect of session (*F*_(1,39)_ = 4.52, *p* = 0.039, *η_p_^2^* = 0.10). Additionally, the right-hand group showed a higher overall percentage of task-classified volumes than the left-hand group, as indicated by a significant main effect of group (*F*_(1,39)_ = 17.24, *p* < 0.001, *η_p_^2^* = 0.31), with a nonsignificant interaction between session and group (*F*_(1,39)_ = 1.78, *p* = 0.19, *η_p_^2^* = 0.04). This significant main effect of group, in the absence of a significant interaction, indicates that the right-hand group exhibited more task-like activation patterns during both pre- and post-RS compared with the left-hand group. This suggests that task-similar activation was present not only during post-RS but already during pre-RS in the right-hand group relative to the left-hand group. The right precentral cortex (Fig. 5*A*, right) showed a marginally significant main effect of group, with higher classification in the left-hand group than in the right-hand group (*F*_(1,39)_ = 4.02, *p* = 0.052, *η_p_^2^* = 0.094). There was no significant main effect of session (*F*_(1,39)_ = 1.94, *p* = 0.17, *η_p_^2^* = 0.047), and a marginally significant interaction was observed (*F*_(1,39)_ = 2.88, *p* = 0.10, *η_p_^2^* = 0.069). The left postcentral cortex (Fig. 5*B*, left) showed significant main effects of both group—higher in the right-hand group than in the left-hand group (*F*_(1,39)_ = 5.61, *p* = 0.023, *η_p_^2^* = 0.13)—and session (*F*_(1,39)_ = 8.95, *p* = 0.005, *η_p_^2^* = 0.19) as well as a significant interaction (*F*_(1,39)_ = 4.64, *p* = 0.04, *η_p_^2^* = 0.11). Post hoc analysis revealed that post-RS had significantly higher classification than pre-RS in the right-hand group (*t*_(20)_ = −3.53, *p* < 0.005, Cohen's *d* = 0.79), whereas no significant difference was observed in the left-hand group (*t*_(19)_ = −0.61, *p* = 0.55, Cohen's *d* = 0.14). The right postcentral cortex (Fig. 5*B*, right) showed no significant main effects of group (*F*_(1,39)_ < 0.001, *p* = 1.00, *η_p_^2^* < 0.001) or session (*F*_(1,39)_ = 2.63, *p* = 0.11, *η_p_^2^* = 0.06), with no significant interaction (*F*_(1,39)_ = 2.12, *p* = 0.15, *η_p_^2^* = 0.05). Although these results were not corrected for multiple comparisons across the four ROIs, they generally indicate a higher frequency of task-like reactivations during the post-task resting period compared with the pre-task period in the left primary sensorimotor cortex, contralateral to the hand used in the right-hand group.

**Figure 6.**
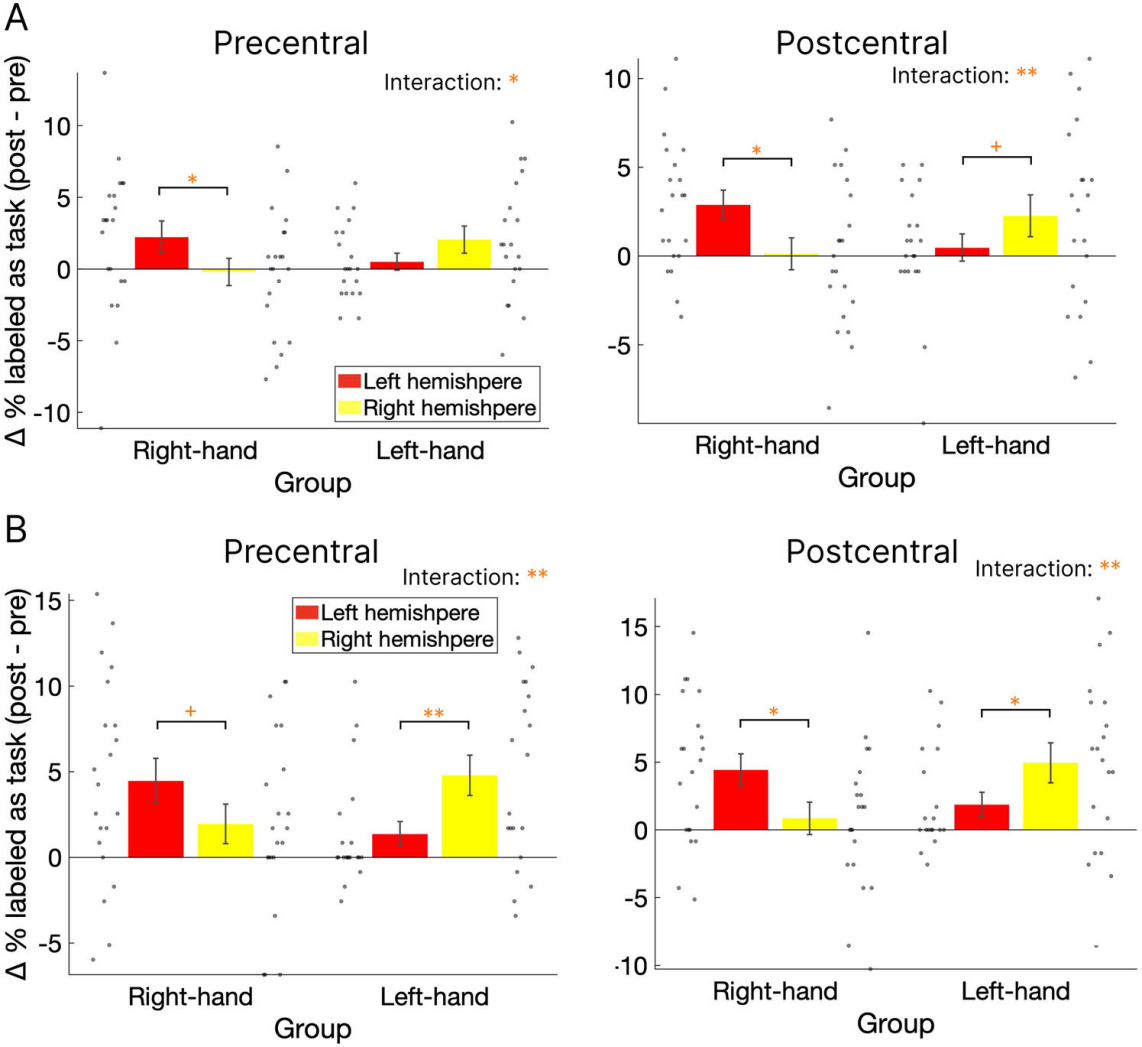
Difference in the percentage of volumes labeled as task patterns between pre-task and post-task rest. Difference in the percentage of volumes labeled as (***A***) or more similar to (***B***) the task pattern relative to the non-task (replay) pattern during pre-RS and post-RS in the precentral and postcentral cortices. Panel ***A*** shows results from the classification analysis; panel ***B*** shows results from the RSA. Red and yellow bars indicate the left and right hemispheres, respectively. Gray dots represent individual participant data. Error bars denote SEMs. ^+^*p* < 0.10; * *p* < 0.05; **p < 0.01. Extended Data Figures 6-1 and 6-2 are supporting Figure 6.

To directly compare differences in classification accuracy between the pre- and post-task periods for each hemisphere and group, we subtracted the pre-RS classification accuracy from the post-RS accuracy using the same results. A mixed-effects ANOVA was conducted on the difference in the percentage of volumes classified as task patterns, with hemisphere (right/left) as a within-subject factor and group (right-hand/left-hand) as a between-subject factor. In the precentral cortex (Fig. 6*A*, left), a significant interaction was observed (*F*_(1,39)_ = 5.93, *p* = 0.02, *η_p_^2^* = 0.12), with no significant main effects of group (*F*_(1,39)_ = 0.07, *p* = 0.80, *η_p_^2^* < 0.001) or hemisphere (*F*_(1,39)_ = 0.30, *p* = 0.59, *η_p_^2^* = 0.008). Post hoc analysis showed a higher percentage of task-labeled volumes in the contralateral hemisphere for both groups, with the difference reaching significance in the right-hand group (*t*_(20)_ = 2.26, *p* = 0.04, Cohen's *d* = 0.51) but not in the left-hand group (*t*_(19)_ = −1.22, *p* = 0.24, Cohen's *d* = 0.28). In the postcentral cortex (Fig. 6*A*, right), there was also a significant interaction (*F*_(1,39)_ = 8.79, *p* = 0.005, *η_p_^2^* = 0.18), with no significant main effects of group (*F*_(1,39)_ = 0.02, *p* = 0.90, *η_p_^2^* < 0.001) or hemisphere (*F*_(1,39)_ = 0.40, *p* = 0.53, *η_p_^2^* = 0.01). Post hoc analysis revealed significantly or marginally significantly greater numbers of task-labeled volumes in the contralateral hemisphere for both the right-hand ((*t*_(20)_ = 2.28, *p* = 0.03, Cohen's *d* = 0.51) and left-hand ((*t*_(19)_ = −1.94, *p* = 0.07, Cohen's *d* = 0.44) groups. Overall, these results demonstrate a higher frequency of reactivations in the primary sensorimotor cortex during the post-task period compared with the pre-task period, with this effect being specific to the left primary sensorimotor cortex contralateral to the hand used in the right-hand group.

10.1523/ENEURO.0134-25.2025.f6-1 Figure 6-1 **Percentage of volumes labeled as task patterns during resting-state (RS) sessions using classifiers trained on individual task sessions** Percentage of volumes in the precentral (A) and postcentral (B) cortices classified as task patterns versus non-task (replay) patterns during the pre-task (pre-RS, blue) and post-task (post-RS, green) RS sessions. Analyses were conducted in the hemisphere contralateral to the hand used. The classifier was independently trained on each of the three task sessions (sessions 1–3). L, left hemisphere; R, right hemisphere. Error bars denote SEMs. Download Figure 6-1, TIF file.

10.1523/ENEURO.0134-25.2025.f6-2 Figure 6-2 **Example of MVPA-decoded labels in the left postcentral cortex of the right-hand group** Frequency of spatiotemporal patterns labeled as task-related during rest, based on MVPA classification (A) and RSA (B). Black labels indicate volumes classified as (A) or more similar to (B) the task pattern compared with the non-task (replay) pattern. In each panel, the top two plots show the task-labeled volumes for each subject (top) and the percentage of task-labeled volumes across subjects (bottom) during the pre-RS session. The bottom two plots show the same data during the post-RS session. Download Figure 6-2, TIF file.

We then performed the same classification analysis of the RS data using a classifier independently trained on each of the three task sessions. This analysis was conducted on the hemisphere contralateral to the hand used (Extended Data Fig. 6-1). A within-subject ANOVA was conducted on the difference in the percentage of volumes classified as task patterns during the resting periods (pre-/post-RS), with the classifier trained separately on Sessions 1, 2, and 3. In the left precentral cortex, a higher percentage of task-classified volumes was observed in post-RS than in pre-RS, as indicated by a significant main effect of resting period (*F*_(1,20)_ = 10.14, *p* < 0.005, *η_p_^2^* = 0.34). There was also a marginally significant main effect of session (*F*_(2,40)_ = 2.90, *p* = 0.067, *η_p_^2^* = 0.13), indicating a trend toward increased task-pattern classification as the sessions progressed, with no significant interaction (*F*_(2,40)_ = 0.04, *p* = 0.96, *η_p_^2^* = 0.002). In the left postcentral cortex, a similar effect was found: a higher percentage of task-classified volumes in post-RS than pre-RS, as indicated by a significant main effect of resting period (*F*_(1,20)_ = 18.40, *p* < 0.001, *η_p_^2^* = 0.48), along with a marginally significant effect of session (*F*_(2,40)_ = 2.62, *p* = 0.085, *η_p_^2^* = 0.12), indicating a trend toward increased task-pattern classification as the sessions progressed, with no significant interaction (*F*_(2,40)_ = 0.56, *p* = 0.57, *η_p_^2^* = 0.03). Similarly, in the right precentral cortex, a higher percentage of task-classified volumes was observed in post-RS than in pre-RS, with a significant main effect of resting period (*F*_(1,19)_ = 18.05, *p* < 0.001, *η_p_^2^* = 0.49) but not of session (*F*_(2,38)_ = 1.30, *p* = 0.29, *η_p_^2^* = 0.06) and no significant interaction (*F*_(2,38)_ = 0.53, *p* = 0.59, *η_p_^2^* = 0.03). In the right postcentral cortex, post-RS showed a higher percentage of task-classified volumes than pre-RS as indicated by a significant main effect of resting period (*F*_(1,19)_ = 12.33, *p* < 0.005, *η_p_^2^* = 0.39), with no significant main effect of session (*F*_(2,38)_ = 1.08, *p* = 0.35, *η_p_^2^* = 0.05) and no significant interaction (*F*_(2,38)_ = 0.44, *p* = 0.65, *η_p_^2^* = 0.02). These results indicate that both the left and right hemispheres showed a significant increase in the frequency of reactivations from pre-RS to post-RS. Additionally, there was a tendency for reactivation to increase across sessions, particularly in the left hemisphere.

We further conducted the same analysis using the RSA results. In the precentral cortex (Fig. 6*B*, left), a significant interaction was observed (*F*_(1,39)_ = 10.23, *p* = 0.003, *η_p_^2^* = 0.21), with no significant main effects of group (*F*_(1,39)_ = 0.01, *p* = 0.91, *η_p_^2^* < 0.001) or hemisphere (*F*_(1,39)_ = 0.23, *p* = 0.63, *η_p_^2^* = 0.005). Post hoc analysis revealed a significantly or marginally significantly higher percentage of task-labeled volumes in the contralateral hemisphere for both the right-hand ((*t*_(20)_ = 1.75, *p* = 0.10, Cohen's *d* = 0.39) and left-hand ((*t*_(19)_ = −3.00, *p* = 0.008, Cohen's *d* = 068) groups. Similarly, in the postcentral cortex (Fig. 6*B*, right), a significant interaction was found (*F*_(1,39)_ = 11.86, *p* = 0.001, *η_p_^2^* = 0.23), with no significant main effects of group (*F*_(1,39)_ = 0.31, *p* = 0.58, *η_p_^2^* = 0.008) or hemisphere (*F*_(1,39)_ = 0.07, *p* = 0.80, *η_p_^2^* = 0.002). Post hoc analysis showed a significantly higher percentage of task-labeled volumes in the contralateral hemisphere for both the right-hand ((*t*_(20)_ = 2.37, *p* = 0.03, Cohen's *d* = 0.53) and left-hand ((*t*_(19)_ = −2.59, *p* = 0.02, Cohen's *d* = 059) groups. These results are consistent with the previous classification analysis, indicating a higher frequency of reactivations in the primary sensorimotor cortex contralateral to the hand used during the post-task resting period.

**Figure 7.**
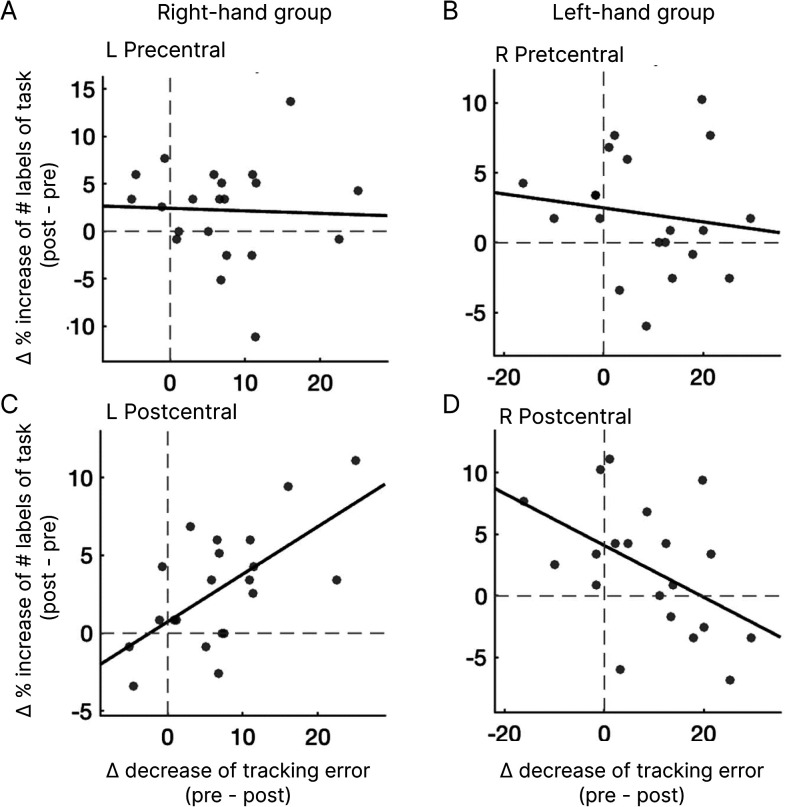
Correlation between MVPA results and behavioral performance. Correlation between the increase in task-pattern labeling (post-RS minus pre-RS) and the reduction in tracking error from pre- to post-task performance. The increase in classification reflects the difference in the percentage of volumes labeled as task patterns in the contralateral precentral and postcentral cortices. L, left hemisphere; R, right hemisphere.

Finally, we investigated the relationship between the frequency of reactivation during rest and behavioral performance. Specifically, we analyzed the correlation between the increase in the percentage of task-labeled volumes from pre-RS to post-RS in the contralateral hemisphere of each group and the decrease in tracking errors from the average of the first three task sessions to the final task session. In the right-hand group, a significant correlation was observed in the left postcentral cortex (*R* = 0.64, *p_Bonf_* < 0.05: Fig. 7*C*), but no significant correlation was found in the left precentral cortex (*R* = −0.04, *p_Bonf_* > 0.05: Fig. 7*A*). In the left-hand group, no significant correlations were found in either the right precentral (*R* = −0.14, *p_Bonf_* > 0.05: Fig. 7*B*) or the right postcentral (*R* = −0.48, *p_Bonf_*  > 0.05: Fig. 7*D*) cortex.

## Discussion

In this study, we used fMRI combined with MVPA to investigate the re-emergence of task-related activation patterns during rest following a visuomotor task. We first classified brain activity patterns from the task and replay (observation-only) blocks using data from the first three task sessions. This analysis showed successful classification, with significantly higher accuracy in the left sensorimotor cortex contralateral to the hand used, compared with the ipsilateral side. Next, we investigated whether task-related activation patterns reappeared more frequently after task training (post-RS) than before the task period (pre-RS). Both multivariate classification and RSA consistently revealed a significantly greater number of reactivations in the primary sensorimotor cortex during the post-task rest period compared with the pre-task rest period. Notably, this effect was robust in the left primary sensorimotor cortex when participants used their right hand and was significantly correlated with motor performance following rest. These findings demonstrate the reactivation of task-related patterns in the primary sensorimotor cortex after a visuomotor task.

Considering the differences between groups, both the left precentral and postcentral cortices of the right-hand group showed a significantly higher percentage of task-labeled volumes in the post-RS compared with the pre-RS, as revealed by both the raw data (Fig. 5) and the direct comparisons (Fig. 6*A*). In contrast, the right precentral and postcentral cortices of the left-hand group showed no significant difference in either analysis (Figs. 5, 6*A*), with a significant effect observed only in the RSA results (Fig. 6*B*). These findings suggest that the observed reactivations are robust but appear to be more prominent and specific to the left hemisphere in the right-hand group. In contrast, reactivation effects in the right hemisphere of the left-hand group were comparatively weaker. Notably, all participants in both groups were right-handed, and the visuomotor task resulted in significantly higher tracking errors in the left-hand group than in the right-hand group. This difference in handedness, or the associated increase in task difficulty, may underlie the hemispheric differences in reactivation observed between groups. Further studies are needed to validate this possibility.

Several previous studies using spatial navigation tasks in rodents have demonstrated that patterns of neural activity associated with spatial experiences are replayed in the hippocampus during rest, contributing to memory consolidation (Euston et al., 2007; Carr et al., 2011; Grosmark and Buzsáki, 2016). In addition to spatial navigation, motor learning has also been shown to induce replay-like activity following training on skilled upper-limb tasks in rodents, which is associated with offline improvements in motor performance (Gulati et al., 2014; Ramanathan et al., 2015). In humans, a recent magnetoencephalography study revealed fast, waking neural replay during rest periods in which rapid consolidation occurs across the hippocampus and neocortex following novel motor sequence learning (Buch et al., 2021). Similarly, a human fMRI study found that multivoxel patterns of RS brain activity corresponded to wrist or finger movements in motor-related areas of both the cerebrum and cerebellum, suggesting that these patterns may be shaped by prior motor experiences (Kusano et al., 2021). Another fMRI study in humans also reported replay activity in the hippocampus during wakeful rest after nonspatial decision-making tasks involving sequential information (Schuck and Niv, 2019), indicating that neural reactivation is a domain-general process and not limited to specific cognitive functions.

A previous study using noninvasive brain stimulation demonstrated that disrupting the primary motor cortex with transcranial magnetic stimulation (TMS) following motor learning leads to a reduction in learning. Notably, this effect was specific to the waking rest period and was not observed during sleep (Robertson et al., 2005). Another TMS study found that disruption of the primary motor cortex impaired early performance gains (early boost) but did not affect delayed gains in motor sequence learning (Hotermans et al., 2008). Together, these brain stimulation studies highlight the role of the primary motor cortex in the offline learning processes involved in visuomotor control.

Regarding the relationship between reactivation and behavior, we found a significant correlation between the frequency of reactivation patterns and the reduction in tracking errors from pre- to post-task in the left postcentral cortex for the right-hand group (Fig. 7*C*), but not in the precentral cortex (Fig. 7*A*). Previous studies have suggested that awake rest following learning supports improvements in behavioral performance during offline visuomotor learning (Brashers-Krug et al., 1996; Shadmehr and Brashers-Krug, 1997; Robertson et al., 2004). This finding—that reactivation in the primary somatosensory cortex correlates with behavioral performance—may suggest that sensory-level reactivation, rather than motor-level reactivation, plays a more critical role in subsequent performance improvements. In contrast, we found no significant correlation in either the precentral or postcentral cortex for the left-hand group (Fig. 7*B*,*D*). As all participants were right-handed, this effect might be influenced by handedness. The left-hand group also showed only nonsignificant or marginally significant increases in task-like volumes during post-RS compared with pre-RS in the precentral and postcentral cortices, respectively (Fig. 6*A*). Moreover, this group exhibited significantly higher tracking errors compared with the right-hand group (Fig. 2), indicating that insufficient learning with the nondominant hand may have contributed to the weaker reactivation effects observed. This possibility warrants further investigation.

The present experiment showed that the left sensorimotor cortex, contralateral to the hand used, exhibited task-like activity compared with the ipsilateral side, not only during the post-task resting period but also during the pre-task resting period. The presence of such task-like activity before the task is somewhat intriguing, but it may be related to the phenomenon of “preplay” observed in the spontaneous neural activity of rodents. Preplay, in contrast to replay, refers to the sequential activation of hippocampal neurons according to future place fields during rest, occurring before the animal actually performs a spatial navigation task (Dragoi and Tonegawa, 2011, 2013). This phenomenon indicates that hippocampal activation during rest may support not only memory consolidation and retrieval but also planning. A recent human fMRI study also reported preplay-like activations during the acquisition of new semantic knowledge (Kurashige et al., 2018). Considering these previous studies, the current finding may similarly reflect preplay-like activation associated with visuomotor skill learning—a possibility that should be investigated in the future.

The current study has some limitations. First, the increased task-like activity observed during the post-task resting period may simply reflect residual brain activity following motor task execution. However, we consider this possibility unlikely for the following reasons. We examined the frequency of volumes classified as task-related by both the classification analysis and the RSA during the resting periods (Extended Data Fig. 6-2). If the observed effect were merely residual brain activity, we would expect a higher concentration of task-like patterns immediately after the task was completed. Instead, the results showed that task-assigned labels were distributed throughout the entire 6 min resting period. This suggests that the observed activity reflects not residual task-related activation, but rather sustained task-related reactivation during rest. Second, related to the previous point, the observed reactivation may reflect that participants were intentionally or unintentionally rehearsing their prior motor experience during rest, despite being instructed to remain at rest (see Materials and Methods). We consider this possibility unlikely, as in such cases, the residual effects of motor memory would be expected to diminish gradually over time. However, as shown above, the proportion of task-like volumes did not decrease over the resting period. Therefore, we consider it unlikely that the observed reactivation reflects rehearsal of motor memory. Nevertheless, further studies employing cognitively demanding tasks during rest are needed to definitively rule out this possibility. Third, the present study examined only a single rotational transformation and did not investigate brain activity specific to different transformation rules. Our previous study demonstrated that multiple rotational transformations can be acquired in the primary sensorimotor cortex (Ogawa and Imamizu, 2013), but the relevance of specific motor skills to the reactivation of brain activity patterns remains unclear in the current study. Lastly, the present study focused only on kinematic motor adaptation involving a rotational transformation, and it remains unclear whether the findings can be generalized to other types of motor tasks, such as sequential motor learning (e.g., the serial reaction time task; Robertson, 2007). This should also be investigated in future studies.

In summary, we found a significant increase in task-related activity during the post-task rest period compared with the pre-task period. This effect was particularly robust in the left primary sensorimotor cortex when the right hand was used and was significantly correlated with motor improvement following rest. These findings demonstrate the reactivation of task-related patterns in the primary sensorimotor cortex after a visuomotor task.

## Synthesis

Reviewing Editor: Christine Portfors, Washington State University Decisions are customarily a result of the Reviewing Editor and the peer reviewers coming together and discussing their recommendations until a consensus is reached. When revisions are invited, a fact-based synthesis statement explaining their decision and outlining what is needed to prepare a revision will be listed below. The following reviewer(s) agreed to reveal their identity: NONE. Note: If this manuscript was transferred from JNeurosci and a decision was made to accept the manuscript without peer review, a brief statement to this effect will instead be what is listed below.

Thank you for addressing all the reviewers' comments.
