# Supplementary figures and images for: Human Sensorimotor Cortex Reactivates Recent Visuomotor Experience during Awake Rest
Source: eNeuro. 2025 Apr 25;12(4):ENEURO.0134-25.2025. doi: 10.1523/ENEURO.0134-25.2025 (PMC12037166; doi:10.1523/ENEURO.0134-25.2025)

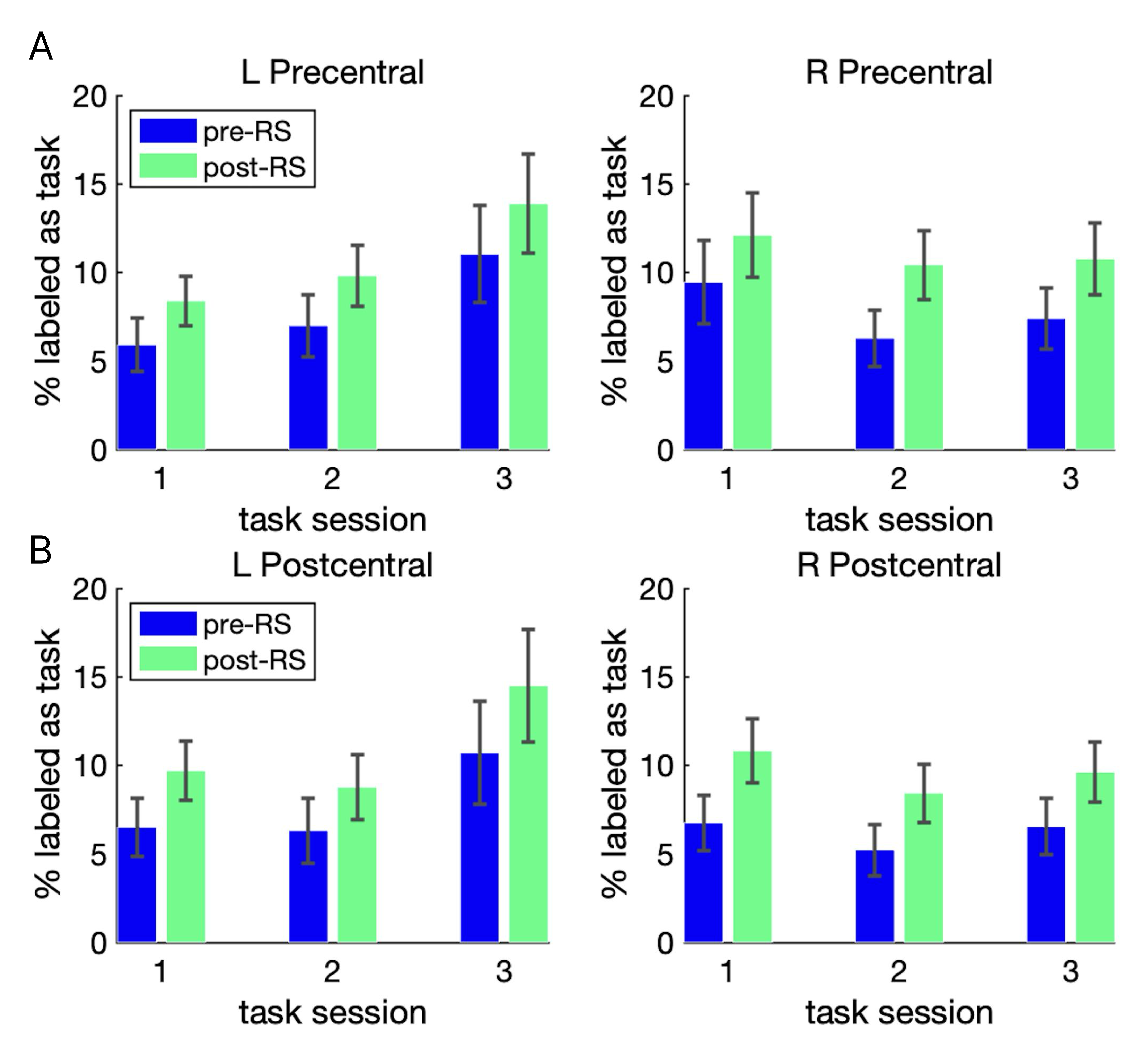

Supplement: Figure 6-1 — Percentage of volumes labeled as task patterns during resting-state (RS) sessions using classifiers trained on individual task sessions Percentage of volumes in the precentral (A) and postcentral (B) cortices classified as task patterns versus non-task (replay) patterns during the pre-task (pre-RS, blue) and post-task (post-RS, green) RS sessions. Analyses were conducted in the hemisphere contralateral to the hand used. The classifier was independently trained on each of the three task sessions (sessions 1–3). L, left hemisphere; R, right hemisphere. Error bars denote SEMs. Download Figure 6-1, TIF file. [file eneuro-12-ENEURO.0134-25.2025-s001.tif]

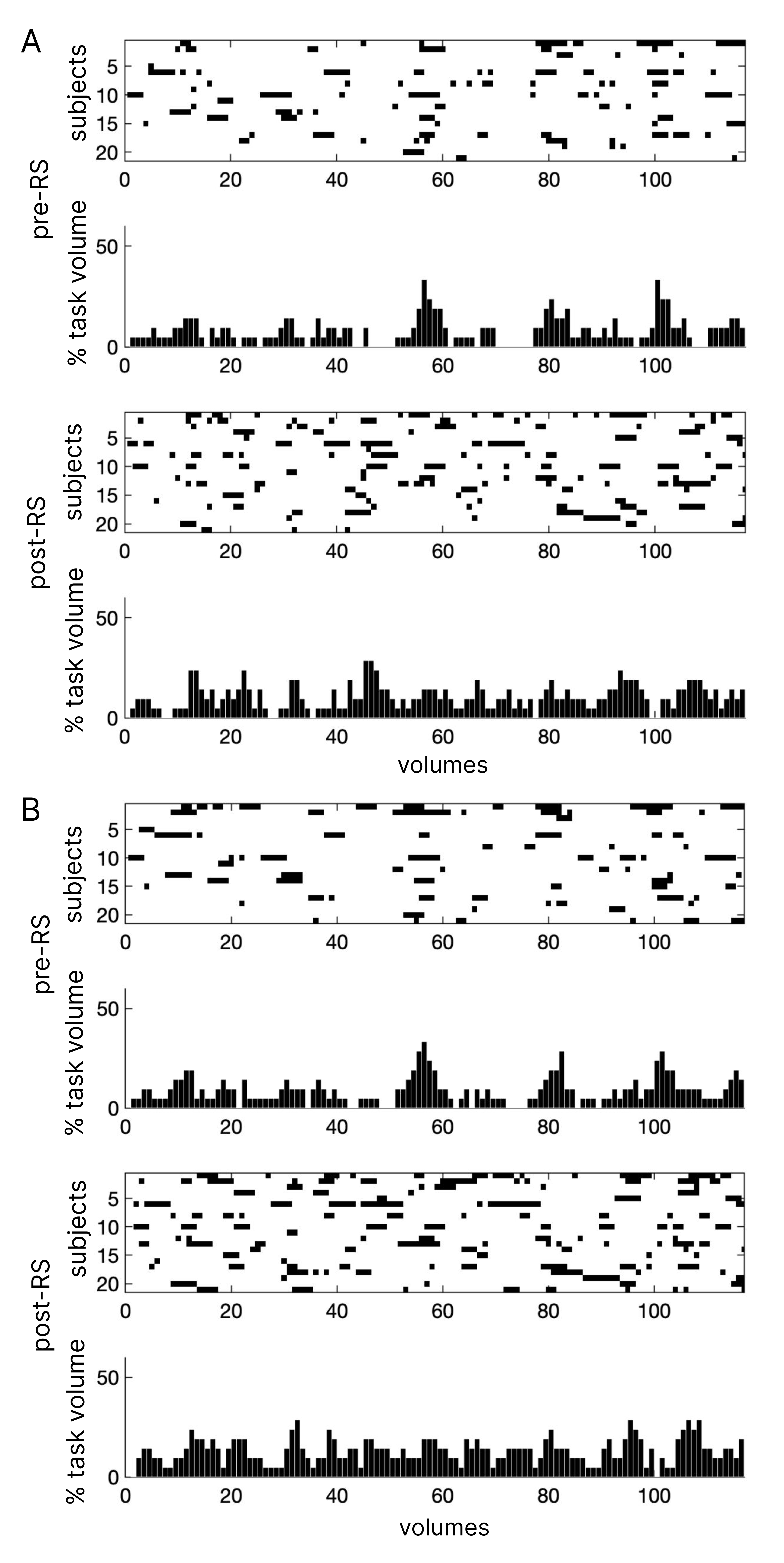

Supplement: Figure 6-2 — Example of MVPA-decoded labels in the left postcentral cortex of the right-hand group Frequency of spatiotemporal patterns labeled as task-related during rest, based on MVPA classification (A) and RSA (B). Black labels indicate volumes classified as (A) or more similar to (B) the task pattern compared with the non-task (replay) pattern. In each panel, the top two plots show the task-labeled volumes for each subject (top) and the percentage of task-labeled volumes across subjects (bottom) during the pre-RS session. The bottom two plots show the same data during the post-RS session. Download Figure 6-2, TIF file. [file eneuro-12-ENEURO.0134-25.2025-s002.tif]
